# *Salmonella* serotypes and their antimicrobial susceptibility in apparently healthy dogs in Addis Ababa, Ethiopia

**DOI:** 10.1186/s12917-017-1055-y

**Published:** 2017-05-19

**Authors:** Bitsu Kiflu, Haile Alemayehu, Mukarim Abdurahaman, Yohannes Negash, Tadesse Eguale

**Affiliations:** 1 Ministry of Livestock and Fisheries, P.O. Box 170042, Addis Ababa, Ethiopia; 2 Aklilu Lemma Institute of Pathobiology, Addis Ababa University, P.O. Box 1176, Addis Ababa, Ethiopia; 3 College of Agriculture and Veterinary Medicine, Jimma University, P.O.Box 307, Jimma, Ethiopia

**Keywords:** *Salmonella*, Serotype, Antimicrobial resistance, Dog, Zoonosis, Addis Ababa, Ethiopia

## Abstract

**Background:**

The close bond between pet animals and family members poses risk of infection with zoonotic bacterial pathogens such as *Salmonella*. No data is available on occurrence of *Salmonella* in dogs in Ethiopia. The aim of this study was therefore to determine the prevalence, serotype distribution and antimicrobial resistance of *Salmonella* from feces of apparently healthy dogs in Addis Ababa, Ethiopia.

**Results:**

Of the total 360 dogs examined, 42 (11.7%; 95% Confidence limit of 8.5%–15.4%) were positive for *Salmonella*. Fourteen serotypes were detected and the predominant ones were *S*. Bronx (*n* = 7; 16.7%), *S*. Newport (*n* = 6; 14.3%), followed by *S*. Typhimurium, *S.* Indiana, *S.* Kentucky, *S*. Saintpaul and *S*. Virchow (*n* = 4; 9.5%) each. *Salmonella* infection status was significantly associated with history of symptom of diarrhea during the past 60 days (OR = 3.78; CI = 1.76–8.13; *p* = 0). Highest resistance rates were found for oxytetracycline (59.5%), neomycin (50%), streptomycin (38.1%), cephalothin (33.3%), doxycycline (30.9%), ampicillin (30.9%) and amoxicillin + clavulanic acid (26.2%). Thirty eight (90.5%) of the isolates were resistant or intermediately resistant to at least one of the 16 antimicrobials tested. Resistance to two or more antimicrobials was detected in 30 (71.4%) of the isolates. Resistance to three or more antimicrobials was detected in 19 (45.2%) of the isolates.

**Conclusion:**

This study demonstrated high carriage rate of *Salmonella* serotypes known for causing human salmonellosis and large proportion of them were resistant to antimicrobials used in public and veterinary medicine for management of various bacterial infections, suggesting the possible risk of infection of human population in close contact with these dogs by drug resistant pathogens. Therefore, it is vital to work on raising public awareness on zoonotic canine diseases prevention measures and good hygienic practices.

## Background

Salmonellosis is an infectious disease of humans and animals caused by several serotypes of *Salmonella*. *Salmonella* is widespread in the environment and commonly found in farm effluents, human sewage and in any material subjected to fecal contamination [1]. *Salmonella* in animals are of major concern, because animals can serve as latent carriers of *Salmonella* serotypes and shed the organism into the environment without any apparent clinical signs posing risk of human infection [2]. Dogs are one of the important asymptomatic carriers of *Salmonella,* as they can harbour large bacterial load in the intestines and mesenteric lymph nodes which can be shed in their feces [3]. This could be of significant importance to public health as dogs have close contact with family members in households.

Antimicrobial resistant *Salmonella* and other zoonotic pathogens originating from companion animals have a great public health importance. The majority of studies on antimicrobial resistance in *Salmonella* focused on food animals and farm environments, since food animals are believed to be the major sources of resistant strains of non-typhoidal *Salmonella*. However, a few studies reported antimicrobial resistant *Salmonella* isolates, including multidrug resistant (MDR) ones from dogs and other companion animals in different parts of the world [4–6].

In Ethiopia, no data is available on occurrence of zoonotic bacterial pathogens in dogs and possible risk of human infection with *Salmonella* from dogs. Majority of previous work focused mainly on food animals and food items [7, 8]. However, there is an increasing trend of keeping dogs in urban areas and demand for general veterinary services for dogs is also increasing [9]. The objectives of this study were therefore to investigate the prevalence, serotype distribution and antimicrobial susceptibility of *Salmonella* isolates from apparently healthy dogs in Addis Ababa, Ethiopia. In addition, various factors were also examined for the possible association of *Salmonella* occurrence in dogs.

## Methods

### Study area and study animals

The study was conducted from January to October, 2015 in Addis Ababa, which is the capital city and administration center for the Federal Democratic Republic of Ethiopia. Among ten sub-cities, four sub-cities namely, Gulele, Arada, Kirkos and Yeka were randomly selected. A total of 360 dogs were involved in this study. Those brought to veterinary clinics for anti-rabies vaccination (*n* = 264) were sampled. In addition, dogs (*n* = 96) were also sampled through door to door visit from households. Apparently healthy dogs of all age groups and both sexes were included in the study. Sick dogs and/or dogs treated recently with antimicrobials were not included in the study.

### Sample and data collection

Rectal swab samples were collected with sterile cotton swab by rotating the swab inside the rectum of the dog and the swab was placed into screw caped test tubes containing 10 ml of sterile buffered peptone water (BPW) (Becton Dickinson, France). The test tubes were properly labeled and transported in ice box to Microbiology Laboratory of Aklilu Lemma Institute of Pathobiology, Addis Ababa University within 3–4 h of collection.

During sample collection, data was also collected from 252 households using a questionnaire that focused on assessing the possible risk factors of *Salmonella* infection. Possible risk factors considered for the presence of *Salmonella* including: age, sex, history of diarrhea during the last 2 months, purpose of dog ownership, type of food and source of food/meat provided to dog were among the questions included in the questionnaire.

### *Salmonella* isolation and identification

Isolation and identification of *Salmonella* was conducted as described previously [7]. Briefly, fecal swabs in buffered peptone water (BPW) pre-enrichment broth were homogenized using vortex mixer for 30 s and incubated at 37 °C for 24 h. A 100 μl pre-enriched suspension was added into 9.9 ml of Rappaport-Vassiliadis enrichment Broth (RVB) (Oxoid, USA) and incubated at 42 °C for 24 h. At the same time, 1 ml of suspension was also transferred to 9 ml of Tetrathionate broth (TTB) (Oxoid, USA) and incubated for 24 h at 37 °C. It was then streaked from both RVB and TTB to Xylose Lysine Tergitol 4 (XLT-4) (Oxoid, USA) and Brilliant Green Agar (BGA) (Difco Becton Dickinson, USA) selective media and the plates were then incubated at 37 °C for 24 h. XLT-4 plates were incubated for additional 24 h and second reading was conducted at 48 h. Presumptive *Salmonella* colonies were further investigated biochemically using Triple Sugar Iron agar, Urea, Citrate and Lysine Iron Agar slants. Those colonies with typical *Salmonella* biochemical properties were then further confirmed by genus specific polymerase chain reaction (PCR) [10]. Reference strain of *S.* Typhimurium (ATCC 14028) was used as a positive control during biochemical analysis and PCR. Confirmed *Salmonella* isolates were stored at −80 °C in 20% glycerol until further testing.

### *Salmonella* serotyping and phage typing

*Salmonella* isolates were serotyped and phage-typed at the Public Health Agency of Canada, World Organization for Animal Health (OIÉ) Reference Laboratory for Salmonellosis, Guelph, Ontario, Canada. Briefly, the somatic (O) antigens were determined by slide agglutination tests [11] and flagellar antigens were determined using a microplate agglutination technique [12]. The antigenic formulae of Grimont and Weill [13] were used to identify and assign the serotypes of the isolates. Phage typing of *S*. Typhimurium isolates was performed according to Anderson et al. [14] with reference phages obtained from the Public Health England, Gastrointestinal Bacteria Reference Unit, Colindale, England and the Public Health Agency of Canada, National Laboratory for Enteric Pathogens, Winnipeg, Canada. *Salmonella* isolates that reacted with the phages but did not conform to any recognized phage type were designated atypical (AT).

### Antimicrobial susceptibility testing

Susceptibility of the isolates to 16 antimicrobials was determined using the disk diffusion method according to the guidelines of the Clinical and Laboratory Standards Institute [15]. Briefly, frozen isolates were subcultured on tryptic soy agar (Becton, Dickinson and Company, USA) from which 3 to 4 pure colonies were inoculated to a tube containing 5 ml of tryptic soy broth (TSB) (Becton, Dickinson and Company, USA) and mixed gently using sterile inoculating loop. It was then incubated at 37 °C for 4–5 h. The turbidity of the suspension was then adjusted to the optical density of a McFarland unit of 0.5 using sterile saline to standardize the inoculum size. Sterile cotton swab was dipped and rotated several times and pressed firmly on the inside wall of the tube above the fluid level to remove excess inoculum. It was then inoculated to Mueller Hinton Agar plate (Oxoid, Ltd) by streaking the swab over the entire surface of the plate. The inoculated plates were left at room temperature to dry for 5–10 min and antimicrobial discs were dispensed by pressing on the plate with sterile forceps and the plates were inverted and incubated at 37 °C overnight. Diameters of the zone of inhibition were measured to the nearest millimeter using a plastic transparent ruler. The interpretation of the categories of susceptible, intermediate or resistant was based on the CLSI guidelines [15]. The cut off points used for the interpretation is shown in Table 1. For the purpose of analysis, all readings classified as intermediate were considered as resistant unless indicated. Reference strain of *Escherichia coli* ATCC 25922 was used as a quality control. The list of antimicrobial discs (Sensi-Discs, Becton, Dickinson and Company, Loveton, USA) used in the study and their strength is shown in Table 1.

**Table 1:** List of antimicrobial discs used in the study, their strength and zone diameter interpretive cut off points in mm

Disk	Disc strength in μg	Resistant	Intermediate	Susceptible
		≤ (mm)	(mm)	≥ (mm)
Amikacin	30	14	15–16	17
Amoxicillin + clavulanic acid	20/10	13	14–17	18
Ampicillin	10	13	14–16	17
Cefoxitin	30	14	15–17	18
Ceftriaxone	30	19	20–22	23
Cephalothin	30	14	15–17	18
Chloramphenicol	30	12	13–17	18
Ciprofloxacin	5	20	21–30	31
Doxycycline	30	10	11–13	14
Gentamicin	10	12	13–14	15
Kanamycin	30	13	14–17	18
Nalidixic acid	30	13	14–18	19
Neomycin	30	12	13–16	17
Oxytetracycline	30	11	12–14	15
Streptomycin	10	11	12–14	15
Sulfamethoxazole and Trimethoprim	23.75 and 1.25	10	11–15	16

### Statistical analysis

The data were computed by using statistical package for social sciences (SPSS version 20.0). The association between *Salmonella* occurrence and pre-specified categorical factors were compared using person’s χ^2^ test. The point prevalence was calculated as the number of infected individuals divided by the number of individual’s sampled × 100. A *p*- value <0.05 was reported as statistically significant.

## Results

### Occurrence of *Salmonella* in dogs

Of the total 360 dogs examined, 42 (11.67%; 95% confidence limit of 8.5%–15.4%) were positive for *Salmonella*. There was no significant difference in prevalence of *Salmonella* among dogs from different sub-cities, purpose for which the dogs were kept and type and source of food/meat provided to dogs. Similarly, there was no significant difference in *Salmonella* carriage among age groups and sex (*p* > 0.05) (Table 2). Isolation of *Salmonella* was observed to be more common among dogs who had diarrhea within the past 2 months compared to those with no history of diarrhea (OR = 3.78, 95% CI = 1.76–8.13) (Table 3).

**Table 2:** Frequency of *Salmonella* carriage among apparently healthy dogs with respect to selected factors

Variables categories		Number examined	No. positive (%)	X^2^(*p*-value)
Sub-cities	Gulele	137	16 (11.7)	2.1 (0.6)
	Arada	78	7 (9)	
	Kirkos	83	13 (15.7)	
	Yeka	62	6 (9.7)	
Sex	Male	291	35 (12.0)	0.2 (0.7)
	Female	69	7 (10.1)	
Age	Puppy (<6 month)	73	11 (15.0)	4.6 (0.3)
	>6 months - 2 yrs	84	9 (10.7)	
	>2 yrs. - 6 yrs	137	16 (11.7)	
	>6 yrs. to 10 yrs	46	2 (4.3)	
	Over 10 yrs	20	4 (20)	
Purpose of dog ownership	Guard	130	19 (14.7)	2.9 (0.2)
	Hobby	86	6 (6.9)	
	Guard + hobby	144	17 (11.8)	
Overall		360	42 (11.7)

**Table 3:** Association of various factors with carriage of *Salmonella* among dogs in Addis Ababa

Variables	Categorical parameter	No. sampled	No. positive for *Salmonella* (%)	OR(95% CI)	X^2^(*p*-value)
Diarrhea during the last months	Yes	62	16 (25.8)	3.8 (1.8–8.1)	(0.0)
	No	190	16 (8.4)		
Type of food provided to the dog	Meat	21	1 (4.8)	-	2.9 (0.3)
	Table scraps	17	4 (23.5)		
	Meat + table scraps	211	27 (12.8)		
	Commercial pet food	3	0		
Source of food/meat for dogs	Local unlicensed markets	198	24 (12.1)	-	0.9 (0.6)
	Licensed butchers	51	8 (15.7)		
	Supermarkets	3	0		
Overall		252	32		

### *Salmonella* serotype distribution

Overall, 14 different *Salmonella* serotypes were recovered, the predominant serotypes were *S.* Bronx (*n* = 7; 16.7%), *S*. Newport (*n* = 6; 14.3%), *S*. Typhimurium (*n* = 4; 9.5%), *S.* Indiana (*n* = 4; 9.5%), *S*. Kentucky (*n* = 4; 9.5%), *S*. Saintpaul (*n* = 4; 9.5%) and *S*. Virchow (*n* = 4; 9.5%). Other serotypes such as *S*. Anatum (*n* = 2), *S*. Haifa (*n* = 2), *S*. Braenderup (*n* = 1), *S*. Chailey (*n* = 1), *S*. Minnesota (*n* = 1), *S*. Muenchen (*n* = 1) and *S*. Tarshyne (*n* = 1) were also identified (Table 4). To our knowledge, *S*. Bronx, *S*. Chailey, *S.* Indiana, *S*. Minnesota and *S*. Tarshyne are reported for the first time in Ethiopia. Phagetyping of the 4 *S*. Typhimurium showed that two of the isolates were phage type 74 while the other two were atypical.

**Table 4:** *Salmonella* serotype distribution and frequency of resistance to various antimicrobials

Antimicrobials tested	*Salmonella* serotypes and No.(%) of isolates ^a^resistant to various antimicrobials (*n* = 42)														
	Bronx	Newport	Indiana	Kentucky	Saintpaul	Typhimurium	Virchow	Anatum	Haifa	Braenderup	Chailey	Minnesota	Muenchen	Tarshyne	No.(%) resistant
	*n* = 7	*n* = 6	*n* = 4	*n* = 4	*n* = 4	*n* = 4	*n* = 4	*n* = 2	*n* = 2	*n* = 1	*n* = 1	*n* = 1	*n* = 1	*n* = 1	
AM	2(28.6)	4(66.7)	1(25)	1(25)	3(75)	-	-	-	1(50)	-	1(100)	-	-	-	13(30.9)
AMC	1(14.3)	4(66.7)	1(25)	1(25)	3(75)	-	-	-	1(50)	-	-	-	-	-	11(26.2)
CF	1(14.3)	4(66.7)	1(25)	1(25)	3(75)	1(25)	1(25)	-	1(50)	-	1(100)	-	-	-	14(33.3)
CRO	-	-	-	-	-	-	-	-	1(50)	-	1(100)	-	-	-	2(4.8)
FOX	1(14.3)	-	1(25)	1(25)	-	-	-	-	1(50)	-	-	-	-	-	4(9.5)
AN	-	-	-	-	-	-	-	-	-	-	-	-	-	-	0
GM	1(14.3)	-	-	-	-	-	-	-	-	-	-	-	-	-	1(2.4)
K	-	-	-	-	-	-	-	-	-	1(100)	-	-	-	-	1(2.4)
N	5(71.4)	3(50)	1(25)	2(50)	2(50)	-	-	2(100)	2(100)	1(100)	1(100)	1(100)	1(100)	-	21(50)
S	3(42.9)	3(50)	4(100)	1(25)	1(25)	1(25)	-	1(50)	1(50)	-	1(100)	-	-	-	16(38.1)
SXT	-	-	1(25)	-	1(25)	-	-	-	1(50)	-	1(100)	-	-	-	4(9.5)
CIP	-	-	-	-	-	-	-	-	-	-	-	-	-	-	0
NA	-	-	1(25)	-	-	-	-	-	-	-	-	-	-	-	1(2.4)
DO	1(14.3)	5(83.3)	1(25)	-	3(75)	1(25)	-	1(50)	1(50)	-	-	-	-	-	13(30.9)
T	3(42.9)	5(83.3)	1(25)	3(75)	4(100)	2(50)	2(50)	2(100)	1(50)	1(100)	-	1(100)	-	-	25(59.5)
C	-	-	1(25)	-	1(25)	-	-	-	-	-	-	-	-	1(100)	3(7.1)

*AN* Amikacin, *AM* Ampicillin, *AMC* Amoxicillin-clavulanic acid, *C* Chloramphenicol, CF Cephalothin, *CRO* Ceftriaxone, *CIP* Ciprofloxcin, *FOX* Cefoxitin, *DO* Doxycycline, *GM* Gentamicin, *K* Kanamycin, *N* Neomycin, *NA* Nalidixic acid, *S* Streptomycin, *SXT* Sulfamethoxazole + Trimethoprim, *T* Oxytetracycline

^a^Isolates intermediately resistant were also considered resistant

### Antimicrobial susceptibility of *Salmonella* isolates

Frequency of isolates resistant or intermediately resistant to various antimicrobials is shown in Table 4. High resistance rate was recorded among isolates to oxytetracycline 25(59.5%), neomycin 21 (50%), streptomycin 16 (38.1%), cephalothin14 (33.3%), doxycycline 13 (30.9%), ampicillin 13 (30.9%), and amoxicillin + clavulanic acid 11(26.2%). All isolates were susceptible to ciprofloxacin and amikacin. Of the 42 *Salmonella* isolates, 38 (90.5%) were resistant to one or more of the antimicrobials tested. Resistance to two or more antimicrobials was detected in 30 (71.4%) of the isolates. Resistance to three or more antimicrobials was detected in 19 (45.2%) of the isolates. Resistance to five or more antimicrobials was detected in 10 (23.8%) of the isolates whereas 4 (9.5%) isolates were MDR to eight or more antimicrobials.

Different serotypes appeared to exhibit disparity in their susceptibility to some of the antimicrobials tested. For instance all *S*. Newport isolates were resistant to three or more antimicrobials. Likewise, 3 of the 4 *S*. Saintpaul isolates were resistant to five or more antimicrobials. On the other hand, strains belonging to *S*. Virchow, *S*. Typhimurium and *S*. Kentucky were resistant to relatively less number of antimicrobials (Table 5).

**Table 5:** Antimicrobial resistance pattern of *Salmonella* serotypes isolated from dogs

Number	Serotypes	No.	Resistance pattern	
			Intermediate	Resistant
1	Anatum	1	N, T	-
2	Anatum	1	N	DO, S, T
3	Braenderup	1	K, N, T	-
4	Bronx	1	N, T	-
5	Bronx	1	T	-
6	Bronx	1	AMC, DO, N, S	AM, CF, FOX, GM
7	Bronx	1	N, T	AM
8	Bronx	2	N, S	-
9	Bronx	1	-	-
10	Chailey	1	N,S	AM, CF, CRO, SXT
11	Haifa	1	N	DO, T
12	Haifa	1	CRO, N, AM	AMC, CF, FOX, S, SXT
13	Indiana	1	N,S	-
14	Indiana	2	S	-
15	Indiana	1	DO, NA, T	AM, AMC, C, CF, FOX, S, SXT
16	Kentucky	1	-	AM, AMC,CF,FOX
17	Kentucky	1	S, T	-
18	Kentucky	2	N, T	-
19	Minnesota	1	N, T	-
20	Muenchen	1	N	-
21	Newport	1	CF, N	AM, AMC, DO, T
22	Newport	1	N	AM, AMC, CF
23	Newport	2	-	DO, S, T
24	Newport	1	AM, N	AMC, CF, DO, S, T
25	Newport	1	DO	AM, AMC, CF, T
26	Saintpaul	1	CF	AM, AMC, DO, T
27	Saintpaul	1	N	AM, AMC, CF, DO, T
28	Saintpaul	1	CF, S	AM, AMC, C, DO, SXT, T
29	Saintpaul	1	T, N	-
30	Tarshyne	1	-	C
31	Typhimurium Pt 74	1	-	-
32	Typhimurium Pt 74	1	CF, S, T	-
33	Typhimurium At	1	-	-
34	Typhimurium At	1	-	DO,T
35	Virchow	2	T	-
36	Virchow	1	-	-
37	Virchow	1	CF	-

## Discussion

Overall *Salmonella* prevalence of 11.7% in the current study is in line with the study conducted in Thailand in dogs which reported 13.2% of *Salmonella* carriage [16]. Similarly, prevalence in dogs in Iran (13.2%), was comparable to the current finding [2]. However, studies in some developed countries showed much lower rates of *Salmonella* carriage compared to the present finding, for example, 0% in Canada [17]; 0.2% in UK [18], 1% in Turkey [19], and 2.3% in Colorado, USA [20]. The possible reason for the high prevalence of *Salmonella* in the current study and other previous studies compared to the ones conducted in developed countries could be due to differences in pet sanitary practices, feeding habit, difference in public awareness about dog zoonosis and socioeconomic status of the owners. Dog owners in developed countries are aware of the importance of hygiene and make use of the available veterinary care for their animals [21]. In addition, some factors such as difference in season of study, geographical areas and isolation methods employed might have also accounted for the observed difference [22]. In the current study, we used rectal swab samples instead of collection of larger volume of fecal samples which could have probably underestimated the true prevalence.

The association between *Salmonella* carriage and history of diarrhea during the past 2 months could presumably be due to *Salmonella* being one of the causes of clinical disease manifested by diarrhea in this dog population. In line to our finding, a study conducted in Texas, USA reported an association between diarrhea and positive *Salmonella* status in dogs [23]. Clinical salmonellosis is rare in dogs, but clinical signs including diarrhea, fever, anorexia, and abdominal pain are not uncommon [24].

A high degree of serotype diversity was observed among *Salmonella* isolates in the present study of which *S*. Bronx, *S*. Chailey, *S.* Indiana, *S*. Minnesota and *S*. Tarshyne had never been reported from Ethiopia. In related studies conducted in other countries, *S.* Newport and *S*. Typhimurium were reported from dogs [25–27] and pet food [28]. Lefebvre et al. [29] reported that *S*. Kentucky and *S.* Typhimurium were the most common serotypes recovered from dogs in Canada. Leonard et al. [30] also reported *S*. Kentucky as the second most dominant serotype isolated from dogs in Ontario, Canada. Some of the *Salmonella* serotypes isolated in the current study such as *S.* Bronx, *S.* Chailey and *S.* Tarshyne had not been previously reported from dogs. However, they were reported from other sources including humans in other countries. *S*. Chailey was isolated from human patients in Korea and New Zeland [31, 32], *S*. Tarshyne from antelope, ostrich and caracal [33]. *S*. Bronx, the predominant serotype in the current study was first isolated from diarrheic human patient in 1955 in USA [34] and our literature search showed no other report from any other source.

Some of the serotypes reported in this study were previously isolated from animals and animal products in the country. For instance: *S*. Newport, *S*.Typhimurium, *S*. Kentucky, *S*. Saintpaul, *S.* Virchow [7, 8, 35]. Moreover, majority of the serotypes identified in the current study such as *S.* Kentucky, *S*. Typhimurium, *S*. Virchow, *S*. Saintpaul were also isolated from diarrheic human patients in Addis Ababa in our recent study [36]. In this study, it was found that stool samples of 7.2% of the diarrheic human patients attending primary health centers in Addis Ababa were positive for *Salmonella*. The co-occurrence of similar serotypes in companion animals, food animals and humans suggests the circulation of these serotypes among various hosts in the study area.

Majority of the isolates in the current study were resistant or intermediately resistant to at least one antimicrobial and the prevalence of resistance was high to oxytetracycline, neomycin and streptomycin. Similarly, previous antimicrobial resistance studies on canine *Salmonella* in Trinidad [22] and Taiwan [37] reported higher levels of resistance. In contrast, a study conducted in Nigeria [38] in *Salmonella* isolates recovered from dogs demonstrated lower resistance rate to most of antimicrobials tested. The reason for difference in antimicrobial resistance profile between studies could be due to difference in serotypes involved and differences in the antimicrobial usage in humans, food animals as well as pets in respective study areas. The one possible reason for higher antimicrobial resistance in our finding could be, as feeding dogs with raw meat is common practice in Addis Ababa, there is a high chance of exposure to antimicrobial resistant *Salmonella* from different animal products used as pet food. High resistance rate to oxytetracycline and streptomycin in this study could be due to the fact that these drugs are the most commonly used antimicrobials in veterinary medicine in the country [7, 39], and as the majority of the dog owners in Addis Ababa feed raw animal products to their dogs [40], chance of being infected with resistant *Salmonella* from these raw animal products is very high. Previous work showed 71.3% of beef obtained from cattle slaughtered in central Ethiopia contained oxytetracycline residue [41]. Uncooked meat products such as head of cattle, sheep and goats, legs of slaughtered animals are the common food given for dogs in Addis Ababa.

*S*. Newport and *S*. Saintpaul showed high rate of resistance to many antimicorbials tested. This may be due to the fact that these serotypes were among the commonly isolated *Salmonella* serotypes from animal products in Ethiopia [42] and they might had possibly been subjected to various antimicrobials which might have rendered them to develop resistace and eventually got access to dogs through food.

## Conclusion

This study showed high carriage rate of *Salmonella* serotypes commonly known for causing human salmonellosis and some of these isolates were resistant to antimicrobials used both in human and veterinary medicine for management of various bacterial infections, suggesting the possible risk of MDR *Salmonella* infection of human population in close contact with these dogs. Therefore, it is important to raise public awareness on zoonotic canine diseases prevention measures and good hygienic practices. Feeding dogs with cooked meat products, provision of clean water and improving hygiene of pet husbandry practices is therefore essential to prevent further spread of *Salmonella* and other foodborne zoonotic pathogens in dogs and people in close contact with dogs.

## Abbreviations

BGA: Brilliant Green Agar
BPW: Buffered peptone water
MDR: Multi-drug resistance
RVB: Rappaport-Vassiliadis Broth
TTB: Tetrathionate broth
XLT-4: Xylose Lysine Tergitol 4
